# The pathological correlation between pulmonary tuberculosis and sarcoidosis patients and the impact of presence of nodules on pulmonary tuberculosis patients

**DOI:** 10.3389/fcimb.2025.1672862

**Published:** 2026-01-14

**Authors:** Yunfeng Sheng, Zhijian Bao, Xiaojing Zhang, Haibo Hua, Yuxin Guo, Wei Gai, Yanfei Cui

**Affiliations:** 1 Tuberculosis Department, Hangzhou Red Cross Hospital, Hangzhou, China; 2 WillingMed Technology Beijing Co., Ltd, Beijing, China

**Keywords:** mycobacterium tuberculosis complex, sarcoidosis, pulmonary microbiota, medication guidance, pulmonary tuberculosis

## Abstract

**Introduction:**

Both pulmonary tuberculosis (PTB) and sarcoidosis (SA) are chronic, systemic, granulomatous diseases. Due to their similar clinical and radiological features, as well as similar pathological characteristics, it is difficult to distinguish. This study aims to explore the pathological correlation between PTB and SA and the impact of nodules formation on the occurrence of PTB.

**Methods:**

We retrospective enrolled 307 patients admitted to the tuberculosis department between January 2022 and March 2024. After applying the inclusion and exclusion criteria, 170 patients were divided into three groups and analyzed: sarcoid tuberculosis group (TB-N, n=59), non-sarcoid tuberculosis group (TB-NoN, n=74), and sarcoidosis group (SA, n=37). Comparative analysis was performed on the clinical characteristics, pathogen profiles, and pulmonary microbial composition differences among the three groups.

**Results:**

Patients in the TB-N and SA group predominantly presented with multiple nodules. Among samples testing positive by both mNGS and conventional microbiological tests (CMT), the proportion of partially matched results was higher in the TB-N group than in the TB-NoN group, with a greater diversity of pathogenic bacteria detected in the TB-N group. ACE index analysis revealed significantly higher microbial richness in the TB-NoN group compared to both SA and TB-N groups. Regarding treatment regimens, combination therapy was more frequently administered in the TB-N group, while single drug treatment predominated in the TB-NoN group. Although the duration of anti-tuberculosis treatment was longer in the TB-N group, this difference did not reach statistical significance.

**Discussion:**

Significant differences in imaging manifestations were observed between TB-N and SA groups. The presence of nodules was associated with a more complex pathogen profile in PTB patients; however, the pulmonary microbial diversity was lower in TB-N than in TB-NoN. PTB patients with nodules predominantly received combination therapy.

## 1 Introduction

Pulmonary tuberculosis (PTB) is a chronic infectious disease caused by *Mycobacterium tuberculosis complex* (MTBC). It ranks as the second leading infectious killer worldwide after COVID-19 and remains a major global cause of morbidity and mortality. PTB can manifest as either primary infection or secondary disease (due to reactivation or reinfection), with its hallmark histopathological feature being caseating nodules. In contrast, sarcoidosis (SA) is a multisystem autoimmune disorder primarily affecting the lungs, characterized pathologically by non-caseating nodules. Due to their overlapping clinical, radiological, and even histopathological features, distinguishing between TB and sarcoidosis poses significant diagnostic challenges (Badar et al., 2011; Mortaz et al., 2016). Most patients with PTB or SA present with non-specific systemic symptoms, such as malaise, generalized fatigue, weight loss, and non-specific fever. Typical pulmonary symptoms seen in PTB patients, including exertional dyspnea, cough, and pleuritic chest pain, can also occur in those with SA. Acute SA may manifest with fever, bilateral hilar lymphadenopathy, and polyarthralgia, which can also be indicative of PTB (Agrawal et al., 2016). The causative pathogen of PTB is MTBC, while significant pathogens associated with infectious sarcoidosis include non-tuberculous *mycobacteria* (NTM) and *Propionibacteriumspecies* (Abe et al., 1984; Esteves et al., 2016). Acid-fast staining of sputum smears is a rapid and widely used diagnostic method for PTB but cannot differentiate between MTBC and NTM (Naoraini Philip and Vanitha Hohn, 2015). The GeneXpert MTB/RIF assay can detect tuberculosis within two hours and improve its diagnostic rate; however, its sensitivity for smear-negative PTB is only about 60%, and it is not applicable for diagnosing NTM infection (Lee et al., 2018). Consequently, for patients with PTB and SA who share similar imaging features, acid-fast staining and GeneXpert alone are insufficient for a definitive differential diagnosis. Moreover, their treatment strategies differ substantially: PTB is managed primarily with anti-tuberculosis drugs, with corticosteroids added only in specific indications; however, SA is treated mainly with corticosteroids and other immunosuppressants (Baughman and Nunes, 2012). Misdiagnosis and inappropriate immunosuppressive therapy in PTB patients can lead to disease transmission, treatment failure, or even fatal outcomes (Wang et al., 2019). Thus, rapid and accurate differentiation between these two diseases is critical for patient prognosis and public health.

This study aims to investigate the pathological correlation between PTB and SA, as well as the impact of nodule formation on PTB progression, by integrating accurate and rapid etiological diagnostic methods with multiple clinical indicators. These indicators include patient symptoms, clinical characteristics, pathogen profiles, and pulmonary microbiota status.

## 2 Materials and methods

### 2.1 Study design and patient enrollment

A retrospective analysis was conducted on the clinical data of 307 patients admitted to the tuberculosis department of Hangzhou Red Cross Hospital between January 2022 and March 2024. The patient inclusion criteria were: (1) age ≥ 18 years; (2) diagnosed with suspected pulmonary tuberculosis (PTB) or sarcoidosis (SA); (3) performed bronchoalveolar lavage fluid (BLAF) mNGS; (4) availability of clinical etiological test results. Patients were excluded based on the following: (1) final diagnosis was neither pulmonary tuberculosis nor pulmonary nodule, despite undergoing BALF mNGS; or (2) having incomplete medical records. Definition of pulmonary nodule: A focal, round or oval, solid or subsolid opacity with increased density compared to the surrounding lung parenchyma, measuring ≤3 cm in maximum diameter on imaging. It may be solitary or multiple, and is not accompanied by atelectasis, hilar lymphadenopathy, or pleural effusion (Gould et al., 2013; Chinese Thoracic Society and Group, 2024).

The diagnosis of tuberculosis was established in all patients based on a composite of clinical, radiographic, and microbiological criteria, with subsequent confirmation by a favorable response to anti-tuberculosis therapy. Sarcoidosis was diagnosed based on ATS/ERS criteria (Crouser et al., 2020; Baughman et al., 2021). For cases with histopathological examination, the diagnosis was confirmed by independent review from experienced pathologists. Cases without histological support were diagnosed via a multidisciplinary team (MDT) consensus. Infectious and other non-infectious granulomatous diseases were rigorously excluded.

Collected clinical data including demographics, laboratory test results, lung imaging results, conventional microbiological detection results and Medication treatment. The performed conventional microbiological testing (CMT) including BALF culture, acid-fast bacteria (AFB) stain, (1-3)-β-D-glucan detection test, galactomannan antigen detection test, T-cell spot test (T-spot), GeneXpert, Respiratory virus PCR and *Cryptococcal* antigen detection.

### 2.2 Clinical metagenomics

Experienced bronchoscopists performed bronchoscopy to obtain BALF specimens. Based on the patient’s chest CT findings, the affected lung regions were selected for bronchoalveolar lavage (BAL). The BAL fluid (3–5 mL) was used for nucleic acid extraction. DNA extraction, library preparation, sequencing, and data analysis methods were performed as described in previous studies (Chen et al., 2023). Pathogen identification was based on RPTM (reads per ten million, the average number of microbe-specific reads per ten million sequencing reads). The positivity thresholds were as follows: For bacteria and fungi, RPTM ≥ 20 (Chen et al., 2023; Jiang et al., 2024); For viruses: RPTM ≥ 3; For specific pathogens (*Cryptococcus* and *Mycobacterium tuberculosis* complex): RPTM ≥ 1 was considered positive (Shi et al., 2020; Liu et al., 2023). Negative controls (nuclease-free water) were used to detection of contamination (Blauwkamp et al., 2019). Bacteria commonly known as skin or respiratory colonizers (e.g., *Corynebacterium*, *Streptococcus*, *Rothia*, and *Lactobacillus*, etc) were categorized as components of the lung’s normal microbiota (Chen et al., 2020; Xie et al., 2021; Shi et al., 2022; Society, 2023; Tan et al., 2023).

### 2.3 Microbial community structure analysis

We calculated the α-diversity indices (including Shannon, Simpson, Chao1 and ACE indices) of microbial community structure, intergroup statistical significant differences were assessed using t-tests. Permutational multivariate analysis of variance (PERMANOVA) was used to test for significant differences of microbial composition between different groups based on the Bray-Curtis dissimilarity algorithm. The LEfSe (Linear Discriminant Analysis Effect Size) method was employed to identify significantly differential biomarkers between different groups. Species having an Linear Discriminant Analysis (LDA) score > 2 were selected as characteristic dominant species for each group. All these analysis were performed with R software (version 4.5.0) using the Vegan, ape, ggplots, ggtree, or microeco package.

### 2.4 Statistics analysis

All statistical analyses were performed using GraphPad Prism 9 (GraphPad Software, Inc.) and SPSS v26.0 (IBM, NY, USA). Normally distributed continuous variables were expressed as mean ± standard deviation (SD), and intergroup differences were analyzed using one-way ANOVA. Non-normally distributed data were presented as median with interquartile range [M (P25, P75)], statistical comparisons among the groups were performed using the Kruskal-Wallis test. When a significant difference was detected, *post hoc* pairwise tests with a Bonferroni adjustment for multiple comparisons were applied. Categorical data were expressed as number and percentage (n, %) and compared between groups using the chi-square test or Fisher’s exact test. A two-tailed *P*-value < 0.05 was considered statistically significant.

## 3 Results

### 3.1 Baseline characteristics of the patients

This study enrolled a total of 307 patients with pulmonary tuberculosis (PTB) or sarcoidosis (SA) who visited the tuberculosis department between January 2022 and March 2024. After applying the inclusion and exclusion criteria, 170 patients were included in the final analysis. Based on the diagnostic criteria, the patients were divided into three groups: the sarcoid tuberculosis group (TB-N, n=59), the non-sarcoid tuberculosis group (TB-NoN, n=74), and the sarcoidosis group (SA, n=37). Analysis of baseline characteristics revealed that the TB-N group had a higher proportion of male patients (79.66%), while the SA group was predominantly female (61.16%) (*P* < 0.001). Cough and expectoration were the most common symptoms, with the highest prevalence in the SA group and the lowest in the TB-N group (86.49% vs. 55.93%, *P* = 0.003; 54.05% vs. 23.72%, *P* = 0.008). Patients in the TB-N and SA group exhibited a higher incidence of multiple pulmonary nodules on imaging (61.02% and 62.16%) <. Pulmonary cavities were observed exclusively in tuberculosis patients (both TB-N and TB-NoN groups). Mediastinal lymphadenopathy was found only in the SA group (*P* < 0.001). Patients in the TB-NoN group demonstrated a higher frequency of mass/patchy opacities (*P* < 0.001). There were no significant differences in age, comorbidities, and immunosuppression among the three patient groups (*P* > 0.05) (**Table 1**).

**Table 1:** The baseline characteristics of the patients in TB-N, TB-NoN and SA group.

Characteristics	TB-N group (n=59)	TB-NoN group (n=74)	SA group (n=37)	*P*-value
Age (mean ± sd)	45 (30-66)	43 (28-67)	54 (40-65)	0.180
Male	47 (79.66%)	49 (66.22%)	14 (37.84%)	<0.001***
Smoking	12 (20.34%)	16 (21.62%)	5 (13.51%)	0.581
Drink	6 (10.17%)	6 (8.11%)	4 (10.81%)	0.873
Comorbidities, n (%)				
Hypertension	10 (16.95%)	10 (13.51%)	7 (18.92%)	0.735
Diabetes	6 (10.17%)	7 (9.46%)	5 (13.51%)	0.801
Malignant tumor	3 (5.08%)	1 (1.35%)	1 (2.70%)	0.447
Immunosuppression	4 (6.78%)	5 (6.76%)	2 (5.41%)	0.957
Symptoms, n (%)				
Fever	6 (10.17%)	8 (10.81%)	4 (10.81%)	0.992
Cough	33 (55.93%)	56 (75.68%)	32 (86.49%)	0.003**
Expectoration	14 (23.73%)	31 (41.89%)	20 (54.05%)	0.008**
Breathlessness	5 (8.47%)	8 (10.81%)	3 (8.11%)	0.859
Hemoptysis	5 (8.47%)	4 (5.41%)	0 (0.00%)	0.281
Chest pain	5 (8.47%)	4 (5.41%)	1 (2.70%)	0.491
Fatigue	2 (3.39%)	1 (1.35%)	0 (0.00%)	0.441
Imaging features, n (%)				
Solitary nodule	23 (38.98%)	0 (0.00%)	14 (37.84%)	<0.001***
Multiple nodules	36 (61.02%)	0 (0.00%)	23 (62.16%)	<0.001***
Cavity	14 (23.73%)	7 (9.46%)	0 (0.00%)	0.002**
Mediastinal lymphadenopathy	0 (0.00%)	0 (0.00%)	10 (27.03%)	<0.001***
Patchy shadow	21 (35.59%)	56 (75.68%)	2 (5.41%)	<0.001***
Bronchiectasis	1 (1.69%)	3 (4.05%)	0 (0.00%)	0.380
Pleural Effusion	1 (1.69%)	2 (2.70%)	0 (0.00%)	0.594
Striated Opacity	2 (3.39%)	1 (1.35%)	1 (2.70%)	0.734
Space-occupying lesion	0 (0.00%)	1 (1.35%)	0 (0.00%)	0.521
Consolidation	1 (1.69%)	3 (4.05%)	0 (0.00%)	0.380

** P<0.01, *** P<0.001.

Analysis of patients’ clinical indicators revealed that the C-reactive protein (CRP) level was significantly higher in TB patients than in SA patients (*P* = 0.012). Although the white blood cell (WBC) count was higher in TB patients compared to SA patients, the difference did not reach statistical significance (*P* = 0.070). Additionally, the creatinine level was higher in the SA group than in the TB group, but this difference was also not statistically significant (*P* = 0.085) (**Table 2**).

**Table 2:** Clinical features of the patients in TB-N, TB-NoN and SA group.

Parameters	TB-N group (n=59)	TB-NoN group (n=74)	SA group (n=37)	*P*-value
Temperature ± (°C)	36.70 ± (36.50, 36.80)	36.70 ± (36.50, 36.80)	36.70 (36.60, 36.80) ±	0.731
Breath ± (times/min)	± 20 (19, 20)	20 (19, 20) ±	20 (19, 20) ±	0.640
WBC (*10^9^/L)	± 6.00 (5.10, 6.70)	± 5.35 (4.40, 7.08)	± 5.00 (4.10, 6.30)	0.070
NEUT (*10^9^/L)	± 3.90 (3.11, 4.56)	± 3.48 (2.72, 4.99)	± 3.40 (2.68, 4.30)	0.218
LY (*10^9^/L)	± 1.10 (0.83, 1.71)	± 1.10 (0.80, 1.41)	1.2 ± 2 (1.00,1.60)	0.394
PLT (*10^9^/L)	± 243.00 (183.50, 282.00)	± 217.50 (183.50, 264.00)	21 ± 6.00 (182.00, 256.00)	0.232
Hb (g/L)	± 128.00 (117.50, 138.50)	± 126.00 (116.25, 136.00)	± 130.00 (117.00, 140.00)	0.399
CRP (mg/L)	23.12 ± 36.34	20.94 ± 31.74	11.95 ± 22.70	0.012*
Cr (μmol/l)	± 73.50 (63.55, 81.85)	± 65.35 (58.10, 79.23)	± 73.00 (62.6, 86.00)	0.085
BUN (mmol/L)	± 4.82 (3.91, 5.81)	± 4.38 (3.61, 5.76)	4.89 (4.19, 6.00) ±	0.365
Bilirubin (μmol/L)	± 10.00 (8.56, 12.55)	± 9.20 (6.93, 12.05)	± 10.00 (7.50, 14.70)	0.272
BG (mmol/L)	± 5.46 (4.84, 6.29)	± 5.20 (4.81, 5.91)	± 5.70 (5.12, 6.20)	0.216

WBC, White blood cell; NEUT, Neutrophil; LY, Lymphocyte; PLT, Platelet; Hb, Hemoglobin; CRP, C-reaction protein; Cr, Creatinine; BUN, blood urea nitrogen; BG, blood glucose.* P<0.005.

### 3.2 The diagnostic performance of MTBC by different methods

**Figure 1:**
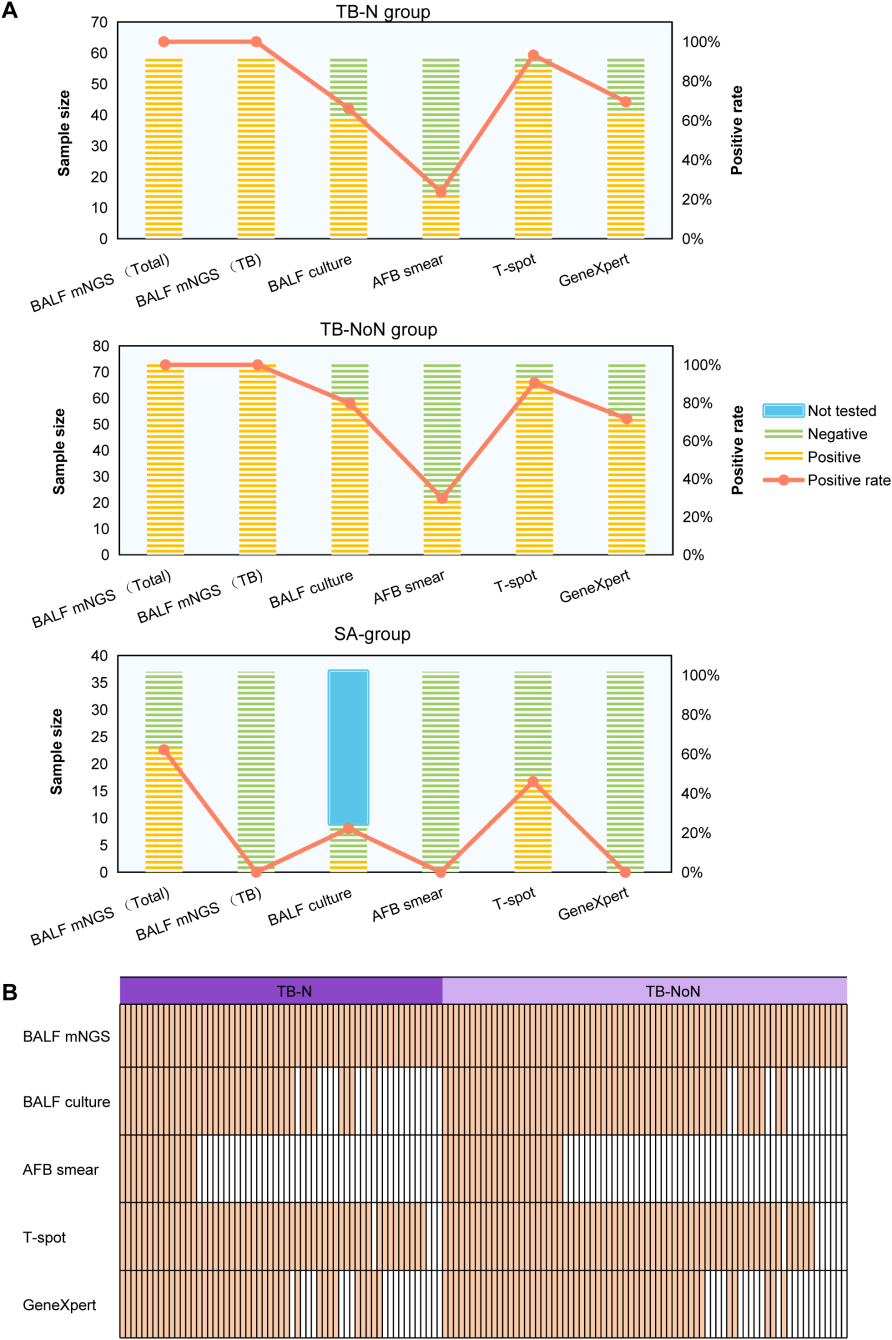
The positive rates of different detection methods and the distribution of MTBC detection. **(A)** The positive rates of different detection methods. **(B)** The detection results of MTBC among patients in the TB-N group and the TB-NoN group by different methods.

We analyzed the pathogen detection results of various methods, including BALF mNGS, BALF culture, AFB smear, T-SPOT, and GeneXpert. The results showed that the positive detection rates of MTBC by BALF mNGS were 100% in both the TB-N and TB-NoN groups, meaning that the overall pathogen detection rate (including MTBC and other pathogens) by BALF mNGS was also 100%. Based on mNGS results, 62.16% of patients in the SA group had detectable pathogens, but none were positive for MTBC. In the TB-N group, the MTBC detection rates followed the order: mNGS (100%) > T-SPOT (93.22%) > GeneXpert (69.49%) > culture (66.10%) > AFB smear (23.73%). While in the TB-NoN group, culture (79.73%) showed a higher positive rate than GeneXpert (71.62%). In the TB-NoN group, the positive rates of MTBC detection by culture, AFB smear and GeneXpert were higher than those in the TB-N group, while T-spot was lower than that in the TB-N group, but the differences were not significant (*P* > 0.05). In the SA group, the BALF culture positivity rate was 22.22% (positive for non-TB pathogenic bacteria), while the T-SPOT positivity rate was 45.95% (**Figure 1A**). The detection situations of various MTBC detection methods in each patient of the TB-N and TB-NoN groups are shown in **Figure 1B**.

**Figure 2:**
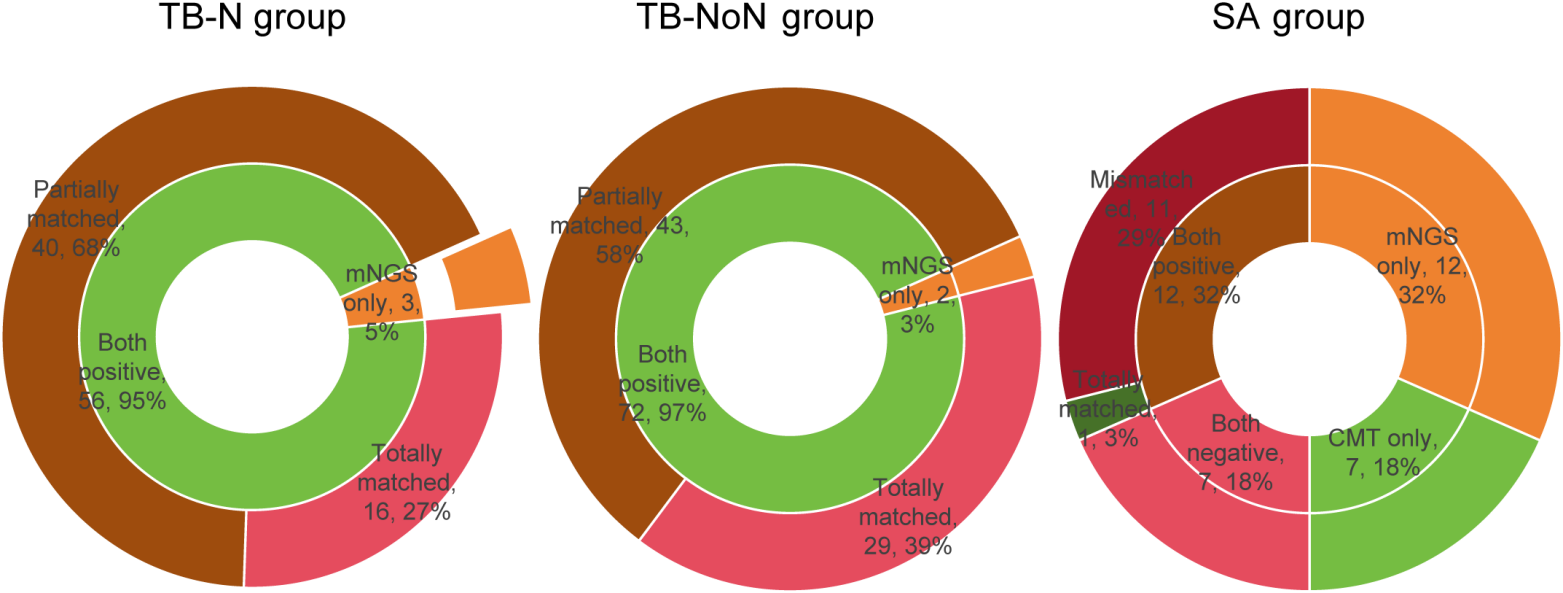
Comparison of the detection consistency of mNGS and CMT in different groups.

We analyzed the concordance of detection between different methods. In the TB-N group, the consistency between mNGS and CMT reached 95%, with the remaining 5% of samples testing positive only by mNGS (**Figure 2**). In the TB-NoN group, the consistency between mNGS and CMT was 97%, with the remaining 3% of samples being mNGS-positive only (**Figure 2**). Compared with the TB-NoN group, more samples in the TB-N group showed partial matches, resulting in additional detection in this group. In the SA group, approximately one-third of patients had double-positive results, primarily due to their non-infectious diagnoses (**Figure 2**).

### 3.3 Pathogen spectrum distribution of patients in different groups

**Figure 3:**
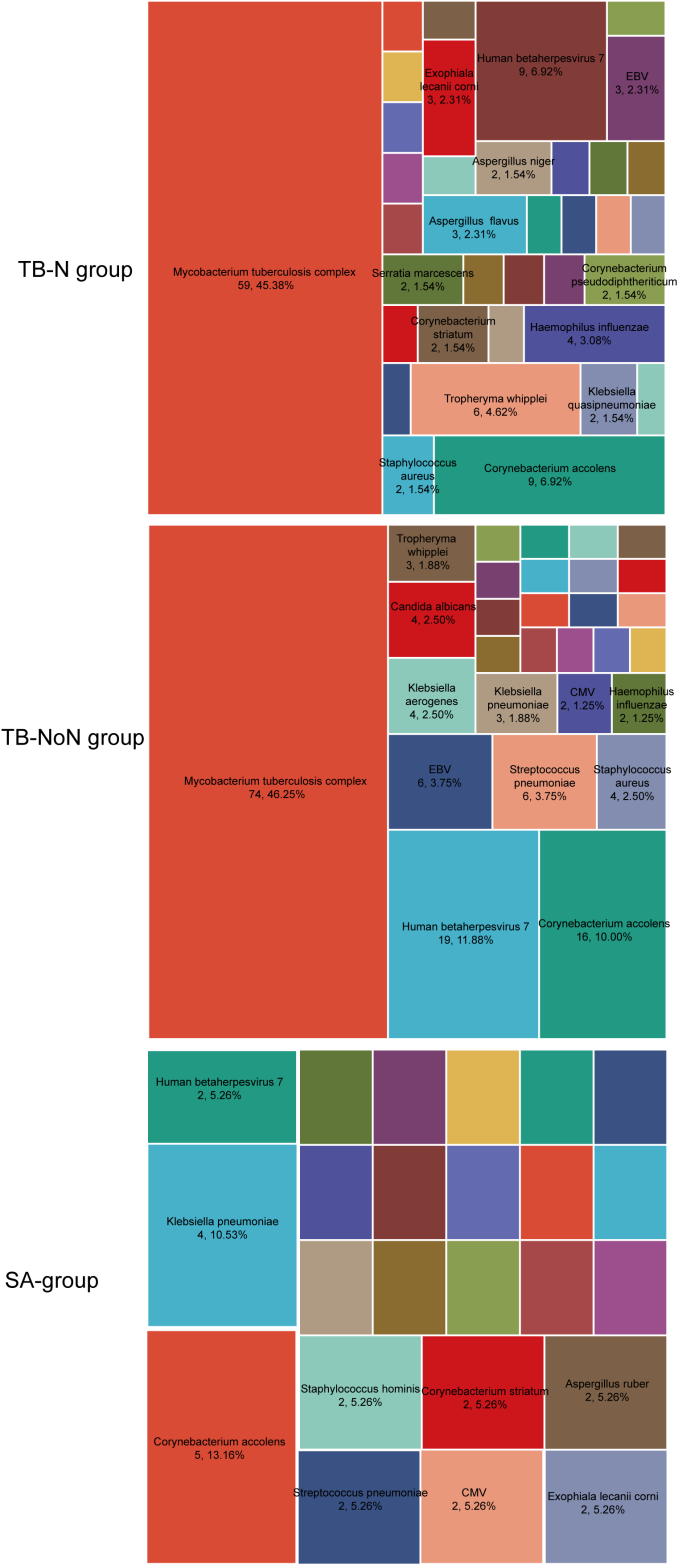
The pathogen spectrum distribution of mNGS detection results.

In both TB-N and TB-NoN groups, MTBC was detected by mNGS in all patients. Notably, the TB-N group demonstrated the highest microbial diversity, with 36 different pathogens identified, predominantly *Corynebacterium accolens*, human herpesvirus 7 (HHV-7), and *Tropheryma whipplei* (**Figure 3**). The TB-NoN group showed 29 detectable pathogens; besides MTBC, the most frequently identified were *C. accolens*, HHV-7, *Streptococcus pneumoniae*, and Epstein-Barr virus (EBV) (**Figure 3**). In the SA group, 24 pathogens were detected, primarily *C. accolens* and *Klebsiella pneumoniae* (**Figure 3**).

### 3.4 The differences in the distribution of pulmonary microbial composition among different groups of patients

**Figure 4:**
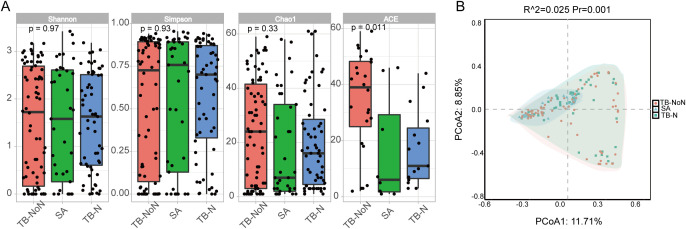
Comparative analysis of the differences in microbial community structure among patients in different groups. **(A)** The α-diversity analysis result. **(B)** The PERMANOVA analysis result.

No significant differences in pulmonary microbial alpha diversity (Shannon and Simpson index) were observed among the different groups. The ACE index revealed a higher microbial richness in the TB-NoN group, and the species numbers in the SA and TB-N groups were significantly lower than those in the TB-NoN group (*P* < 0.05) (**Figure 4A**). PERMANOVA results revealed significant differences in microbial community structure among the three groups (*P* = 0.001). Specifically, significant differences were observed between both the TB-N and TB-NoN groups when compared to the SA group (*P* = 0.0015), whereas no significant difference was detected between the TB-N and TB-NoN groups themselves (*P* = 0.176) (**Figure 4B**).

**Figure 5:**
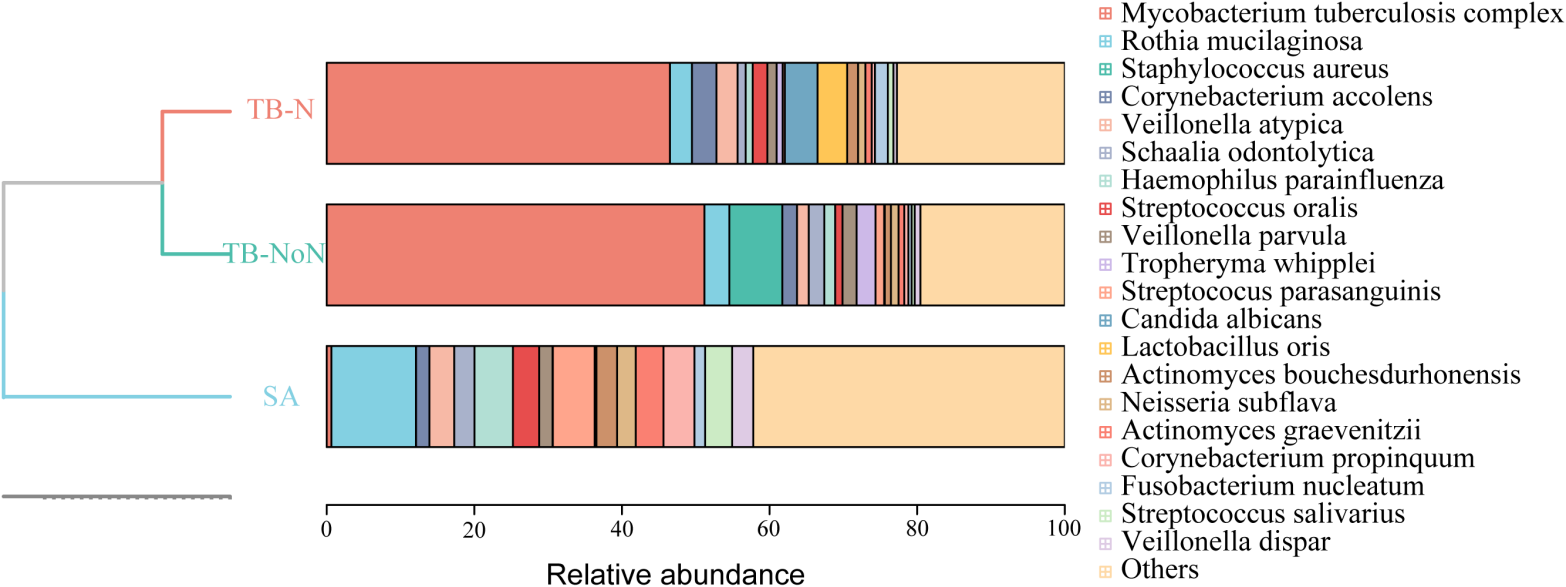
The distribution of pulmonary microbial composition among patients in different groups.

In both the TB-N and TB-NoN groups, samples showed better clustering in the phylogenetic tree. The most frequently detected pathogens in the TB-N group were the MTBC, *Staphylococcus aureus*, and *Rothia mucilaginosa*. In contrast, the TB-NoN group was predominantly characterized by MTBC, *Candida albicans*, and *Lactobacillus oris*. Meanwhile, the SA group most commonly exhibited *Rothia mucilaginosa*, H*aemophilus parainfluenzae*, and *Streptococcus parasanguinis* (**Figure 5**).

**Figure 6:**
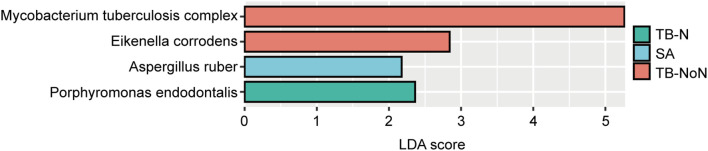
LefSe differential species analysis.

LEfSe analysis was performed to identify representative species across different groups. Based on LDA scores, the microbial biomarker for the TB-NoN group was *Eikenella corrodens* (besides the MTBC). The TB-N group’s biomarker was *Porphyromonas endodontalis*, while the SA group’s biomarker was *Aspergillus ruber* (**Figure 6**).

We further explored the potential correlations between the microbial findings with the clinical data. Spearman correlations between α-diversity indices (including the Simpson index, Shannon index, and Chao index) and patients’ clinical indicators showed that age, presence of diabetes, fever, and solitary nodule showed significant negative correlations with the Chao1 and Shannon indices; Hb was significantly positively correlated with Chao1; and multiple nodules were significantly positively correlated with the Simpson index (**Supplementary Figure 2**). We further applied the multivariate statistical test Adonis to identify the impacts of clinical factors with significant intergroup differences to the community structure. The analysis revealed that only age had a significant effect, explaining 0.96% of the variation (F = 1.625, P = 0.025) (**Supplementary Table 2**).

### 3.5 The clinical medication situation and its impact on the prognosis of patients

**Figure 7:**
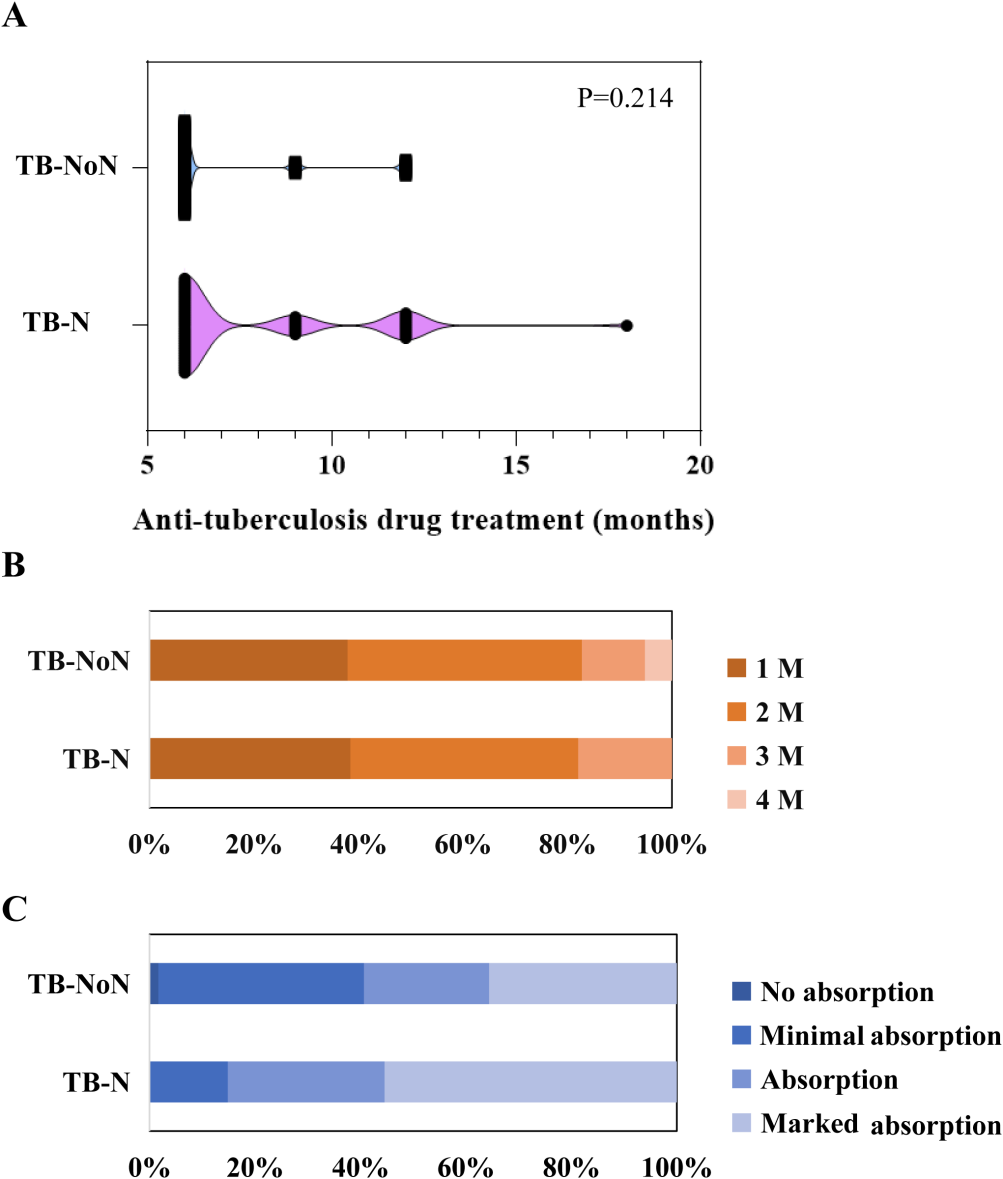
Comparative analysis of the duration of anti-tuberculosis treatment **(A)**, sputum conversion **(B)** and radiologic resolution **(C)** in patients of the TB-N group and the TB-NoN group.

Analysis of the clinical medication guidance based on mNGS and CMT results revealed that all patients in the TB-N and TB-NoN groups underwent anti-tuberculosis (TB) treatment adjustments based on mNGS findings, while over 90% received medication modifications guided by CMT. In the SA group, untreated patients were primarily non-infectious cases. Within the TB-N group, a higher proportion of patients received combination therapy with multiple drug types, whereas the TB-NoN group predominantly received single-agent anti-TB treatment. All anti-TB medications used were first-line agents, including isoniazid, rifampicin, ethambutol, and pyrazinamide. No significant difference was observed in the number of patients receiving anti-TB drugs between the TB-N and TB-NoN groups (**Table 3**). Although anti-TB treatment duration was longer in the TB-N group, the difference did not reach statistical significance (**Figure 7A**). The sputum conversion rate was 100% in all TB-NoN (16 missing) and TB-N (20 missing) patients. The time to sputum conversion ranged from 1 to 4 months, but no significant difference was observed between the two groups (**Figure 7B**). Radiologic resolution was graded as follows: no absorption, minimal absorption (<1/3 absorption), moderate absorption (1/3–50% absorption), and marked absorption (>50% absorption). A significant difference was found between the two groups (*P* = 0.004). Specifically, the TB-N group had a significantly higher proportion of minimal absorption compared to the TB-NoN group (*P* < 0.05), but a significantly lower proportion of marked absorption (*P* < 0.05) (**Figure 7C**). Recurrence occurred in 3 patients (4.05%) in the TB-NoN group, with no significant difference in recurrence rates between the groups. All patients survived at 90 days after discharge. Further analysis was conducted to determine whether these factors, mycobacterial load, and pathogen types detected by mNGS affect the pulmonary microbiome composition in TB-N patients. The results indicated that the level of mycobacterial load is a significant influencing factor (**Supplementary Figure 3**).

**Table 3:** Clinical medication situation.

Medication Information	TB-N group (n=59)	TB-NoN group (n=74)	SA group (n=37)	*P*-value
Antibiotic Adjustment Based on mNGS Results	59 (100.00%)	74 (100.00%)	1 (2.70%)	<0.001
Antibiotic Adjustment Based on CMT Results	56 (94.92%)	69 (93.24%)	0 (0.00%)	<0.001
Combination Drug Therapy (Bacterial, Fungal, Viral, or Tuberculosis Agents)	33 (55.93%)	26 (35.14%)	0 (0.00%)	<0.001
Single Drug Type	26 (44.07%)	48 (64.86%)	5 (13.51%)	<0.001
Anti-Tuberculosis Drugs				
Isoniazid	59 (100.00%)	74 (100.00%)	—	>0.999
Rifampicin	50 (84.75%)	61 (82.43%)	—	0.853
Ethambutol	57 (96.61%)	68 (91.89%)	—	0.300
Pyrazinamide	51 (86.44%)	60 (81.08%)	—	0.485
Antibacterial Drugs				
Cefmetazole	6 (10.17%)	10 (13.51%)	1 (2.70%)	0.201
Moxifloxacin	8 (13.56%)	4 (5.41%)	1 (2.70%)	0.094
Cefoxitin	6 (10.17%)	7 (9.46%)	1 (2.70%)	0.380
Cefperazone-Sulbactam	5 (8.47%)	3 (4.05%)	1 (2.70%)	0.384
Levofloxacin	5 (8.47%)	4 (5.41%)	1 (2.70%)	0.491
Latamoxef	2 (3.39%)	2 (2.70%)	0 (0.00%)	>0.999
Azlocillin	2 (3.39%)	1 (1.35%)	0 (0.00%)	0.584
Meropenem	1 (1.69%)	0 (0.00%)	0 (0.00%)	0.388
Linezolid	0 (0.00%)	1 (1.35%)	0 (0.00%)	0.521
Antifungal Drugs				
Voriconazole	4 (6.78%)	0 (0.00%)	0 (0.00%)	0.020
Fluconazole	0 (0.00%)	1 (1.35%)	0 (0.00%)	0.521

## 4 Discussion

According to WHO data, a total of 1.25 million people died from TB in 2023. After trailing behind COVID-19 for three years, TB may have once again become the world’s leading cause of death from a single infectious agent. It is estimated that approximately one-quarter of the global population is infected with MTBC, with about 5%–10% of infected individuals eventually developing symptoms and progressing to active TB (Global Tuberculosis Report 2024). Global Burden of Disease (GBD) 2021 data indicate an increasing worldwide burden of pulmonary nodules. The overlapping clinical and radiological manifestations of TB and SA pose challenges in differential diagnosis (Gupta et al., 2012). Therefore, this study aims to investigate the pathological correlation between PTB and SA, as well as the impact of nodules on PTB patients, by establishing an accurate and rapid etiological diagnostic approach. This will be combined with multiple indicators, including patient symptoms, clinical characteristics, pathogen profiles, and pulmonary microbial community status.

Most patients with TB or sarcoidosis present with nonspecific systemic symptoms such as fatigue, generalized weakness, weight loss, and low-grade fever. Classic pulmonary symptoms typically seen in TB patients - including exertional dyspnea, cough, and pleuritic chest pain - may also occur in sarcoidosis. Acute sarcoidosis patients may develop fever, bilateral hilar lymphadenopathy, and polyarthralgia, which can also be observed in TB (Agrawal et al., 2016). Tuberculosis typically presents primarily with respiratory symptoms such as cough and sputum production. Systemic manifestations like fever, fatigue, and weight loss occur less frequently in patients with limited pulmonary lesions, unless complicated by concurrent infections or febrile conditions such as rheumatological diseases (Van’t Hoog et al., 2022). Therefore, the findings of this study are consistent with clinical observations. Our study revealed that cough and expectoration were the most prevalent symptoms among both PTB and SA patients, occurring in 71.18% of cases, with the highest incidence observed in SA patients (86.49%). In contrast, fever and fatigue were less common, present in only 10.59% and 1.76% of patients, respectively (**Table 1**).

Microbiological detection of MTBC serves as a crucial method for identifying PTB. In this study, culture, AFB smear, T-SPOT, GeneXpert, and mNGS were employed for MTBC diagnosis. The results demonstrated the following diagnostic capabilities for MTBC (excluding mNGS): T-SPOT > culture > GeneXpert > AFB smear (**Figure 1**). Notably, T-SPOT showed positive results not only in PTB patients but also in some SA cases, indicating that relying solely on T-SPOT for TB diagnosis may lead to misdiagnosis. Previous studies have identified MTBC as the causative pathogen of tuberculosis, while infectious sarcoidosis is primarily associated with non-tuberculous mycobacteria (NTM) and *Propionibacterium* species (Abe et al., 1984; Esteves et al., 2016). Our study revealed that some TB patients had co-infections with viruses or fungi, predominantly *Corynebacterium accolens*, human herpesvirus 7, *Streptococcus pneumonia*e, and *Tropheryma whipplei*. In contrast, SA patients more frequently exhibited *Corynebacterium accolens* and *Klebsiella pneumoniae* (**Figure 3**). This difference might be caused by regional variations, or it could be due to the fact that this study employed more sensitive and comprehensive high-throughput sequencing technology covering a wider range of pathogens. Beyond microbiological tests, imaging examinations play a vital role in TB diagnosis, particularly for sputum-negative cases, patients unable to produce sputum, or suspected extrapulmonary TB (EPTB). Conversely, sarcoidosis diagnosis primarily relies on imaging findings or lung biopsy results (Gould et al., 2013). Therefore, understanding the differences in pulmonary imaging between TB and sarcoidosis patients is essential for differential diagnosis. Our imaging results showed that while nodules were present in both TB-N and SA groups, multiple nodules predominated in TB-N patients (61.02%) whereas solitary nodules were more common in SA patients (62.16%) (**Table 1**). Additionally, pulmonary cavities were exclusively observed in TB patients (both TB-N and TB-NoN), mediastinal lymphadenopathy occurred only in sarcoidosis patients, and mass/patchy shadows were more frequently seen in TB-NoN patients (**Table 1**).

Recent studies have revealed complex interactions between the pulmonary microbiome and various respiratory diseases, with disease states significantly altering patients’ lung microbial composition (Yi et al., 2022; King, 2024). Our findings demonstrated no significant differences in Simpson and Shannon indices among the different groups, indicating comparable community diversity of lung microbiota across groups. However, ACE index analysis showed significantly greater microbial quantity enrichment in the TB-NoN group, with both SA and TB-N groups exhibiting markedly lower species counts compared to TB-NoN - reflecting substantial differences in community richness (**Figure 4**). ACE is an important indicator for assessing microbial species richness. Previous studies have found that, compared to healthy controls, tuberculosis patients exhibit a significant increase in lung microbial richness (ACE and/or Chao1) (Du et al., 2022; Sun et al., 2025). In the present study, the SA group had the lowest pathogen positivity rate, with only 13.51% having used antibiotics, suggesting minimal influence from infection. Their lung microbial composition was relatively similar to that of healthy individuals. This may be an important reason why the ACE index in the TB-NoN group was significantly higher than that in the SA group. Notably, the TB-NoN group displayed significantly higher MTBC abundance than the TB-N group (**Figure 5**). There were no studies similar to ours have been identified that examine differences in lung microbial composition between tuberculosis patients with and without nodules. Whether this trend is typical requires confirmation with a larger sample size. Representative species across groups included MTBC, *Eikenella corrodens*, *Porphyromonas endodontalis*, and *Aspergillus ruber* (**Figure 6**). *Eikenella corrodens* is part of the oropharyngeal, upper respiratory tract, and mucosal microbiota and is considered an opportunistic pathogen capable of causing various infections, primarily in the head and neck region. Most infections are mild and benign; however, under specific conditions, it can lead to severe invasive infections. It has been reported to cause liver abscesses and endocarditis, among other conditions (Nordholm et al., 2018; Li et al., 2022). Currently, there are no reports on the pathogenicity of *Aspergillus ruber*.

This study has several limitations that should be acknowledged. First, as a single-center investigation conducted over a 2.5-year period, while the overall sample size was moderately sized, the findings may still be subject to potential selection bias. Second, all TB-N group participants in this study were non-sarcoidosis patients. The inclusion of cases with concurrent sarcoidosis and tuberculosis might have yielded more representative and clinically relevant results. This study suggests that the diagnosis of tuberculosis requires comprehensive interpretation using multiple microbiological methods. Following larger-scale studies, it would be feasible to develop a predictive model for disease severity by integrating clinical symptoms, radiographic findings, microbiological results, and lung microbiome composition.

In conclusion, our study revealed significant differences in pulmonary imaging findings between the TB-N and SA groups, which may establish an important basis for distinguishing these two populations - specifically, TB-N patients predominantly exhibited multiple nodules, whereas SA patients primarily presented with solitary nodules. Furthermore, the differences in lung microbial richness and representative species between the TB-NoN and TB-N groups suggest that the presence of nodules may reduce the diversity of pulmonary microbial composition in PTB patients. Additionally, the fact that PTB patients with nodules require additional non-tuberculosis medications implies that nodule formation may exacerbate the infectious state in these patients.

## Data Availability

The data presented in the study are deposited in the NCBI SRA database, accession number was PRJNA1393781.
