# Supplementary material for: The pathological correlation between pulmonary tuberculosis and sarcoidosis patients and the impact of presence of nodules on pulmonary tuberculosis patients
Source: Front Cell Infect Microbiol. 2026 Jan 14;15:1672862. doi: 10.3389/fcimb.2025.1672862 (PMC12847324; doi:10.3389/fcimb.2025.1672862)
Supplement: Supplementary file 1 [file Table1.docx]

Supplementary Material

## Supplementary Figures

##
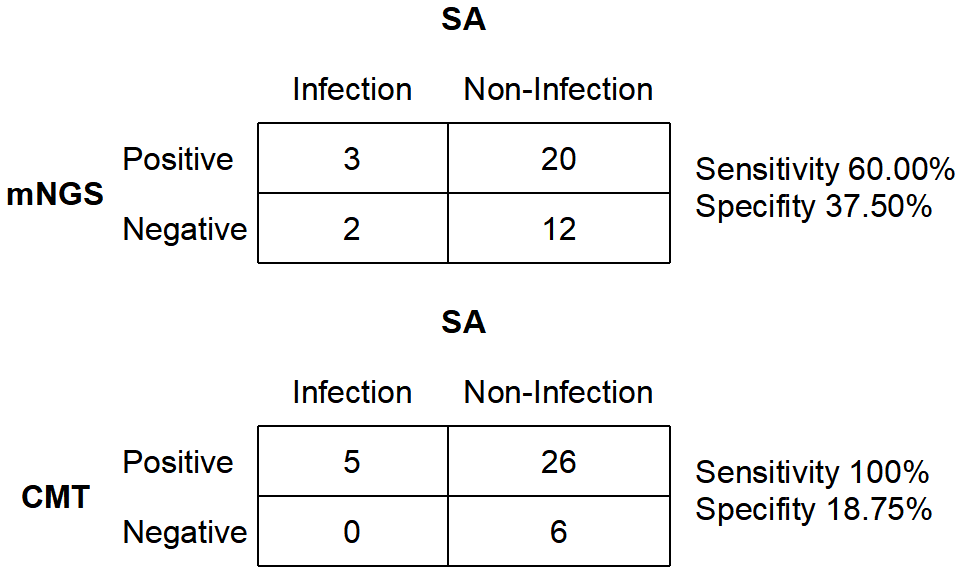


## Supplementary Figure 1. Contingency tables for mNGS and CMT results vs. diagnosed infected SA.

##
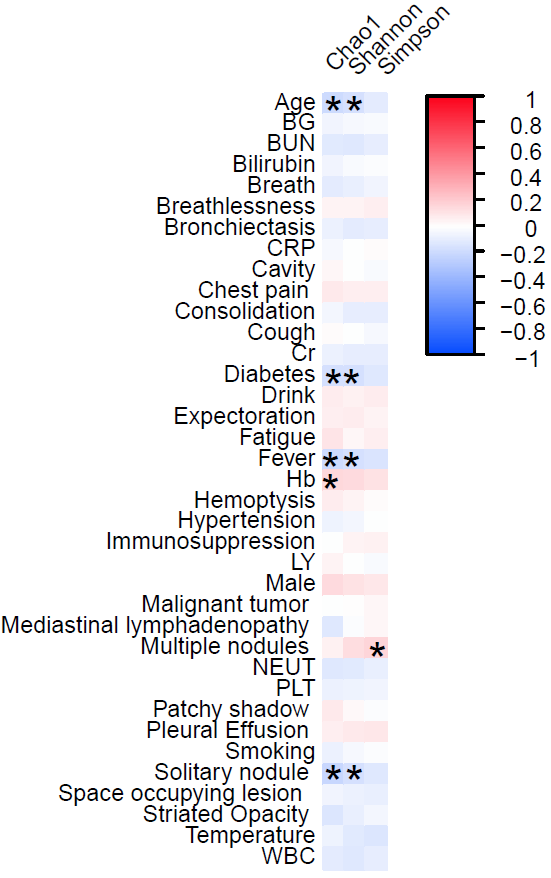


## Supplementary Figure 2. Spearman correlation analysis was performed to assess the relationship between microbial alpha diversity and clinical indicators.


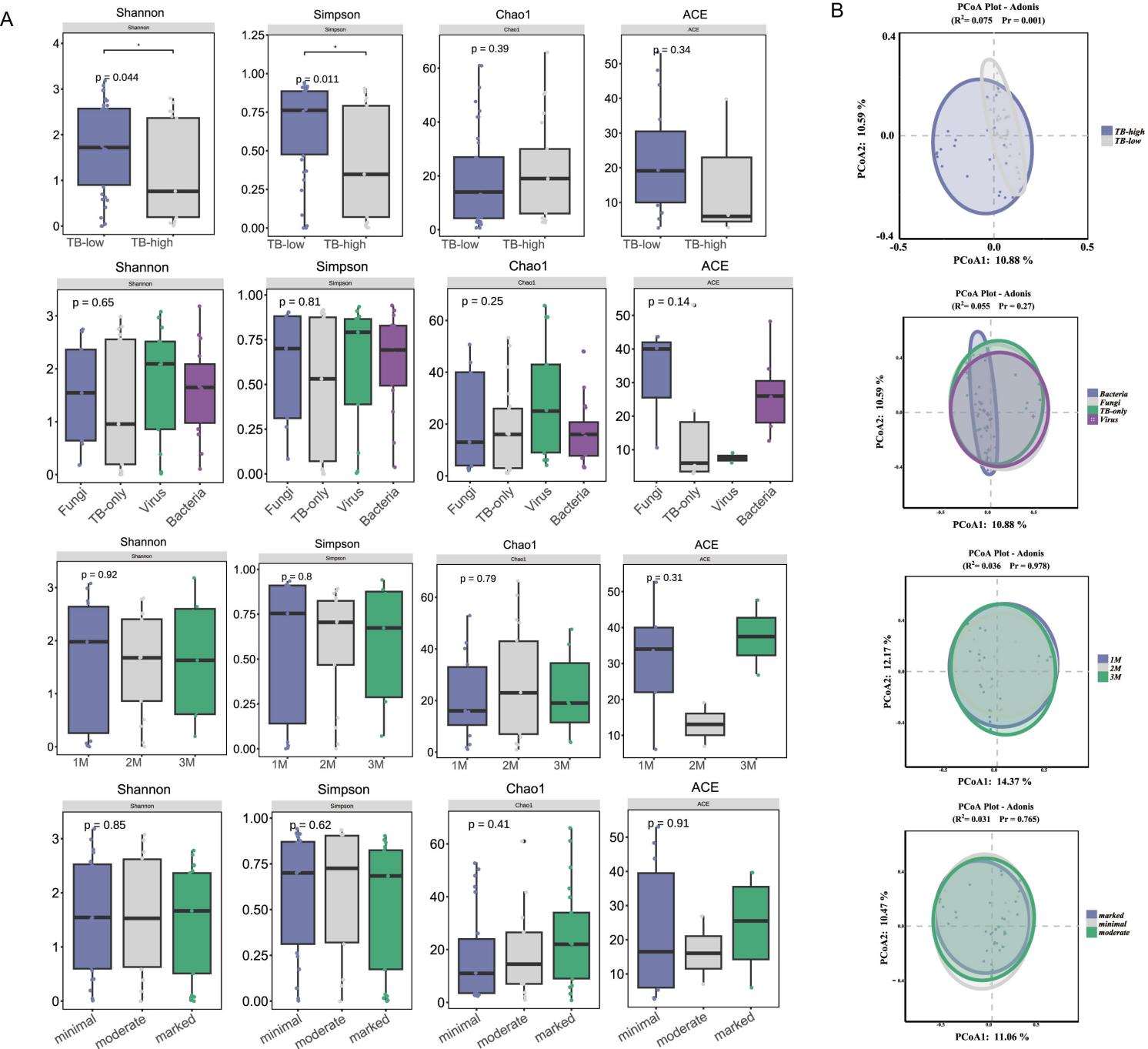


Supplementary Figure 3. Analysis of microbiome composition changes in different subgroups of TB-N patients. Grouped based on tuberculosis abundance: TB-low (RPTM=1–999) and TB-high (RPTM≥1000, n=21); pathogen types detected by mNGS: TB-only, Bacteria (samples with bacteria other than tuberculosis detected, but no fungi or viruses), Fungi (including samples with bacteria and fungi detected, and samples with only fungi detected), and Virus (including samples with bacteria and viruses detected, fungi and viruses detected, and only viruses detected); time to sputum conversion (months): 1M, 2M, 3M; imaging changes, according to lesion absorption: minimal absorption (<1/3 absorption), moderate absorption (1/3–50% absorption), and marked absorption (>50% absorption).

## Supplementary Tables

Supplementary Table 1. Results of multiple comparisons for indicators with significant differences.

| **Characteristics** | **TB-N group (n=59)** | **TB-NoN group (n=74)** | **SA-group (n=37)** | P-value | **Adjust P-value** | | |
| --- | --- | --- | --- | --- | --- | --- | --- |
|  |  |  |  |  | **TB-NoN** | **SA** | **TB-N** |
| **Male** | 47 (79.66%) | 49 (66.22%) | 14 (37.84%) | <0.001*** | a | b | a |
| **Symptoms, n (%)** |  |  |  |  |  |  |  |
| Cough | 33 (55.93%) | 56 (75.68%) | 32 (86.49%) | 0.003** | a | a | b |
| Expectoration | 14 (23.73%) | 31 (41.89%) | 20 (54.05%) | 0.008** | a,b | b | a |
| **Imaging features, n (%)** |  |  |  |  |  |  |  |
| Single nodule | 23 (38.98%) | 0 (0.00%) | 14 (37.84%) | <0.001*** | a | b | b |
| Multiple nodules | 36 (61.02%) | 0 (0.00%) | 23 (62.16%) | <0.001*** | a | b | b |
| Cavity | 14 (23.73%) | 7 (9.46%) | 0 (0.00%) | 0.002** | a,b | b | a |
| Mediastinal lymphadenopathy | 0 (0.00%) | 0 (0.00%) | 10 (27.03%) | <0.001*** | a | b | a |
| Patchy shadow | 21 (35.59%) | 56 (75.68%) | 2 (5.41%) | <0.001*** | a | b | c |
| **Clinical test** |  |  |  |  |  |  |  |
| CRP (mg/L) | 23.12±36.34 | 20.94±31.74 | 11.95±22.70 | 0.012* | a | b | a |
| **Medication Information** |  |  |  |  |  |  |  |
| Antibiotic Adjustment Based on mNGS Results | 59 (100.00%) | 74 (100.00%) | 1 (2.70%) | <0.001 | a | b | a |
| Antibiotic Adjustment Based on CMT Results | 56 (94.92%) | 69 (93.24%) | 0 (0.00%) | <0.001 | a | b | a |
| Combination Drug Therapy (Bacterial, Fungal, Viral, or Tuberculosis Agents) | 33 (55.93%) | 26 (35.14%) | 0 (0.00%) | <0.001 | a | b | c |
| Single Drug Type | 26 (44.07%) | 48 (64.86%) | 5 (13.51%) | <0.001 | a | b | c |
| Voriconazole | 4 (6.78%) | 0 (0.00%） | 0 (0.00%) | 0.02 | a | a | a |

Supplementary Table 2. A multivariate statistical test (Adonis) was applied to evaluate the extent to which clinical indicators with significant differences explain the variation in beta diversity (community structure).

| **Factors** | **R^2^** | **F-value** | **P-value** |
| --- | --- | --- | --- |
| Age | 0.00960 | 1.6254 | 0.0249 |
| Cough | 0.00497 | 0.8408 | 0.7106 |
| Expectoration | 0.00397 | 0.6727 | 0.9483 |
| Solitary nodule | 0.00805 | 1.3630 | 0.0885 |
| Multiple nodules | 0.00479 | 0.8111 | 0.7722 |
| Cavity | 0.00579 | 0.9812 | 0.4722 |
| Mediastinal lymphadenopathy | 0.00637 | 1.0780 | 0.3228 |
| Patchy shadow | 0.00789 | 1.3366 | 0.1026 |
| CRP | 0.00539 | 0.9124 | 0.5991 |
| Anti TB time | 0.00499 | 0.8446 | 0.7116 |
| Anti TB drug | 0.00595 | 1.0082 | 0.4211 |
| Anti bacteria drug | 0.00461 | 0.7801 | 0.8234 |
| Anti fungal drug | 0.00524 | 0.8877 | 0.6540 |
| Residual | 0.90356 |  |  |
